# Nitrate transformation and immobilization in particulate organic matter incubations: Influence of redox, iron and (a)biotic conditions

**DOI:** 10.1371/journal.pone.0218752

**Published:** 2019-07-05

**Authors:** Fiona R. Kizewski, Jason P. Kaye, Carmen Enid Martínez

**Affiliations:** 1 Department of Ecosystem Science and Management, The Pennsylvania State University, University Park, PA, United States of America; 2 Soil and Crop Sciences, School of Integrative Plant Science, Cornell University, Ithaca, NY, United States of America; University of La Frontera, CHILE

## Abstract

Nitrate can be reduced to other N inorganic species via denitrification and incorporated into organic matter by immobilization; however, the effect of biotic/abiotic and redox condition on immobilization and denitrification processes from a single system are not well documented. We hypothesize nitrate (NO_3_^-^) transformation pathways leading to the formation of dissolved- and solid-phase organic N are predominantly controlled by abiotic reactions, but the formation of soluble inorganic N species is controlled by redox condition. In this study, organic matter in the form of leaf compost (LC) was spiked with ^15^NO_3_^-^ and incubated under oxic/anoxic and biotic/abiotic conditions at pH 6.5. We seek to understand how variations in environmental conditions impact NO_3_^-^ transformation pathways through laboratory incubations. We find production of NH_4_^+^ is predominantly controlled by redox whereas NO_3_^-^ conversion to dissolved organic nitrogen (DON) and immobilization in solid-phase N are predominantly controlled by abiotic processes. Twenty % of added ^15^N-NO_3_^-^ was incorporated into DON under oxic conditions, with abiotic processes accounting for 85% of the overall incorporation. Nitrogen immobilization processes resulted in N concentrations of 4.1–6.6 μg N (g leaf compost)^-1^, with abiotic processes accounting for 100% and 66% of the overall (biotic+abiotic) N immobilization under anoxic and oxic conditions, respectively. ^15^N-NMR spectroscopy suggests ^15^NO_3_^-^ was immobilized into amide/aminoquinones and nitro/oxime under anoxic conditions. A fraction of the NH_4_^+^ was produced abiotically under anoxic conditions (~10% of the total NH_4_^+^ production) although biotic organic N mineralization contributed to most of NH_4_^+^ production. Our results also indicate Fe(II) did not act as an electron source in biotic-oxic incubations; however, Fe(II) provided electrons for NO_3_^-^ reduction in biotic-anoxic incubations although it was not the sole electron source. It is clear that, under the experimental conditions of this investigation, abiotic and redox processes play important roles in NO_3_^-^ transformations. As climatic conditions change (e.g., frequency/intensity of rainfall), abiotic reactions that shift transformation pathways and N species concentrations from those controlled by biota might become more prevalent.

## Introduction

Nitrogen (N) is an essential nutrient that exists within a tight global cycle. Increased anthropogenic additions (e.g., NO_3_^-^) have however resulted in excess N that short-circuits this natural cycle; the consequences of this short-circuiting (i.e., bypass) are pollution of our air and water resources. Nitrate can be reduced to other N inorganic species via denitrification (NO_2_^-^, NO, N_2_O, N_2_) and incorporated into organic matter by immobilization (Org-N). Immobilization is understood as a mechanism of NO_3_^-^ transformation that entails the reduction of NO_3_^-^ possibly to more reactive NO_2_^-^ before immobilization into organic N. The formation of organic-N species can potentially prevent NO_3_^-^ leaching losses and the production of N_2_O, a potent greenhouse gas, while retaining N within ecosystems in chemical forms that are available for plant and microbial uptake. Although abiotic pathways for the formation of N trace gases are relatively well recognized [1–4], abiotic pathways and the extent of these chemical processes to the formation of dissolved and solid-phase organic-N species is still subject to debate. Expected changes in climatic conditions (e.g., frequency and intensity of precipitation that affects redox gradients and microbial growth rates and metabolism) may increase the prominence and occurrence of abiotic transformations. Since anoxic/suboxic conditions are likely to increase the concentration of species presumed active in abiotic N cycling (e.g., NO_2_^-^, Fe^2+^, reduced forms of organic matter), studies focused on abiotic pathways and redox conditions, and their contribution to the global N cycle are warranted [5].

Studies on forest ecosystems show that soils, rather than plants, are the primary sink for applied inorganic N (N_i_) [6,7]. Nitrogen retention in soils is largely attributable to N immobilization, a process that converts N_i_ to organic nitrogen (N_o_) [8–10]. Huygens et al. (2008) [11] demonstrated that, one day after addition to volcanic soils, 50% and 60% of the NH_4_^+^ and NO_3_^-^ were retained in solid organic matter, respectively. It has been generally accepted that N immobilization is primarily driven by microbial activity [12,13]. Yet, N immobilization can occur very rapidly (within minutes to hours), so rapidly that it cannot be explained solely by microbial activity [14–16]. In addition, CO_2_ respiration (a consequence of organic carbon mineralization), often deemed necessary to fuel microbial N immobilization [17], was not detected during the time when N_i_ was rapidly immobilized [18]. The implication of these findings is that N_i_ can be immobilized to N_o_ via abiotic processes as well. In the past decades, evidence supporting abiotic N immobilization has been accumulating [15,19–23]. However, contrasting results have also been reported. Colman et al. (2007) [24] found little N was retained in any of the 44 sterilized soils amended with NO_3_^-^. The controversy on abiotic N immobilization exemplifies that our understanding of this process is still insufficient; much remains to be learned about the magnitude and mechanisms that control abiotic N immobilization.

Nitrate is the most mobile N species in soils, likely due to the lack of non-bonding electrons in its electronic structure. In comparison, nitrite (NO_2_^-^), which has a pair of non-bonding electrons, is expected to have a stronger ability to bind to organic matter. Nitrogen in NO_3_^-^ is in its highest oxidation state (5+). NO_3_^-^ transformation to N_o_ involves a decrease in N oxidation state due to the formation of C-N bonds. Therefore, it has been hypothesized that NO_3_^-^ is first reduced to NO_2_^-^ (3+ oxidation state) prior to immobilization and that ferrous iron (Fe(II)) can serve as an electron donor in NO_3_^-^ reduction [23,25]. If this theory holds, anoxic conditions found in the interior of soil aggregates, flooded soils, low oxygen zones in streams, or wetlands are expected to facilitate NO_3_^-^ immobilization. However, when a soil’s redox condition is conducive to NO_3_^-^ reduction, the reduction may not be arrested at NO_2_^-^ formation but may continue to produce gaseous N species (e.g., N_2_) by microbial denitrification or NH_4_^+^ by dissimilatory nitrate reduction to ammonium (DNRA) [26–29]. In addition, reducing conditions may facilitate anaerobic ammonium oxidation (anammox). All these processes can lead to NH_4_^+^ production and/or to gaseous N loss to the atmosphere. As a result, immobilization may be competing with these NO_3_^-^ attenuation processes for the available N under anoxic conditions. Currently, there is insufficient information to enable evaluation of the influence of redox conditions on NO_3_^-^ immobilization, particularly abiotic immobilization.

While some studies have shown that NO_3_^-^ was largely retained in SOM [11,21], rapid NO_3_^-^ transformation to DON has also been observed [15,20,23,30]. In comparison with NO_2_^-^ and NH_4_^+^, NO_3_^-^ was found more prone to conversion to DON. Both Dail et al. (2001) [15] and Fitzhugh et al.(2003) [20] found the amount of NO_2_^-^ retained in SOM was more than five times that of NO_3_^-^. Nitrate transformation to DON potentially explains the unrecovered N in ^15^N tracer studies and N loss from ecosystems [31]. Indeed, after investigating more than 100 streams, Perakis and Hedin (2002) [32] concluded that DON loss from unpolluted forests was the primary source for N input to nearby streams. Yet it is, again, largely unclear how environmental variables regulate this process. It has been suggested that NO_3_^-^ conversion to DON occurs via abiotic reactions [15,23,33,34] but well-designed abiotic experiments are required to verify the involvement of microbial activity in NO_3_^-^ transformation to DON.

Whether N_i_ transformations lead to the formation of dissolved- and solid-phase organic N, the molecular structures of N_o_ and the influence of N_o_ on the storage and release of this essential nutrient into plant-available forms are largely unknown [35,36]. Recent studies on the characterization of N_o_ forms have found pyridines, anilides, amines, and amides are important components of soil and humic acid N [37–42]. Furthermore, the identity of these dissolved and solid-phase molecular structures is important in N biological availability, and in N retention/mobility and accumulation in soils. For example, anilide-N structures have been postulated to result in decreased N availability and reduced crop (rice) yields as these structures are presumed to be less easily mineralized into available forms [43] than more labile N forms such as amino N [35]. Identification of N_o_ molecular structures could therefore lead to a better understanding of the reactions that result in the formation of stable N [44].

Although strictly abiotic conditions are not likely to exist in soils, abiotic (micro)sites prevail due to heterogeneous distribution of microbial colonies [45]. In a broader environmental context, NO_3_^-^ and leaf-derived organic matter interact in oxic and anoxic conditions in streams, wetlands, sediments, and organic layers of forest soils. Nitrate transformations in these environmental settings are expected to be controlled by both biotic and abiotic processes; thus it is important to understand the circumstances under which these processes govern the fate of NO_3_^-^. The existing disparate results on abiotic N immobilization and the uncertainty of how redox conditions affect N immobilization justify more in-depth investigations into NO_3_^-^ transformations under different environmental conditions. In the present study, we investigate how variations in biotic-abiotic and oxic-anoxic conditions influence the dynamics of NO_3_^-^ transformations when ^15^N-NO_3_^-^ is added to leaf compost and incubated in an aqueous suspension for 5 days. We hypothesize nitrate (NO_3_^-^) transformation pathways leading to the formation of dissolved- and solid-phase organic N are predominantly controlled by abiotic reactions, whereas the formation of soluble inorganic N species is controlled by redox condition. Our experimental set up permits answers to questions such as: which nitrate transformation processes are predominantly controlled by redox condition and which are predominantly controlled by biota? In particular, we define the redox condition that exert the greatest influence on N immobilization and the significance of abiotic N immobilization. In addition to the magnitude of N immobilization, we attempted to identify molecular structures of solid-phase N species resulting from N immobilization using ^15^N-NMR spectroscopy. The experiments we conduct provide a comprehensive assessment of macroscopic processes and are critical to understanding the character of N stored in terrestrial ecosystems and ultimately the release of this essential nutrient into bioavailable forms. Knowledge of the environmental conditions that lead to specific N transformation products is important to understanding controls of nutrient losses that will improve water and air quality.

## Materials and methods

### Incubation materials and experimental set-up

The organic matter used in this study is a sugar maple (*Acer saccharumm L*.) leaf compost (LC) collected from a rural location near Ithaca, NY [46]. The LC was air-dried, ground and sieved to obtain the size fraction <1 mm. The LC contains 374 g OC kg^-1^ and 19.4 g N kg^-1^. The LC was rinsed twice with deionized water and then with 0.01 M KCl to remove residual N before oven-drying at 60°C overnight. Each incubation was conducted in a LC suspension prepared by mixing the solid material (2 g) with 500 mL of 0.01 M KCl background electrolyte solution. A nitrate solution (50 mM) was made with 98% ^15^N enriched K^15^NO_3_ (Sigma Aldrich, U.S.A.) and 2 mL of this solution was spiked into the LC suspension to obtain a NO_3_^-^ concentration of 200 μM and ^15^N input of 750 μg ^15^N g^-1^ LC. Leaf compost suspensions without NO_3_^-^ addition were also included and represented blank incubations. The suspensions (experimental and blank) were incubated for five days under four sets of conditions: biotic-oxic (Oxic), abiotic-oxic (γ-Oxic), biotic-anoxic (Anox), and abiotic-anoxic (γ-Anox).

The reaction vessel containing the LC suspension had two openings through which a pH and an E_h_ electrode were inserted, in addition to ports for sampling and gas dispersion. The pH electrode was connected to an automatic acid/base titrator to achieve a constant pH of 6.5. Anoxic conditions (E_h_ ~ -330 mV) were attained by passing a constant N_2_ gas flow into the vessel. For abiotic incubations, the LC was sterilized by gamma (γ) irradiation (6 Mrad dose) at the Breazeale Nuclear Reactor, The Pennsylvania State University. To test the sterility of the γ-irradiated LC, 0.1 g of material was incubated in 25 ml of peptone-tryptone-yeast extract-glucose (PTYG) growth media at 25°C for two weeks. The incubated PTYG growth media (0.1 ml) was then evenly plated onto an R-2A filled cell culture dish, which was then incubated at 25°C for two weeks. At the end of the incubation, microbial colonies were not detected under a microscope. Appropriate aseptic techniques were used during all abiotic incubations. At the end of an experimental γ-Oxic incubation, the LC suspension was subjected to a most probable number (MPN) analysis with 2-fold dilution and five replicates. The experimental result was 1-0-0-0, indicating an estimated cell count of 0.2 cells/ml suspension or 50 cells g^-1^ LC (approximately 10^6^ to 10^9^ cells g^-1^ live soil) [47], thus confirming sterility was well maintained throughout the entire incubation period. During the incubations, aliquots of the suspensions (~25 ml) were withdrawn each day and filtered with 0.2 μm membranes to separate solutions from solid materials. Solutions were frozen immediately for future analyses while the solids were air-dried and then oven dried at 40°C for 6 h. Experimental and blank incubations, each under the four set of conditions, were conducted twice and the data reported are averages of duplicate experiments.

### Analytical methods

Concentrations of NO_3_^-^+NO_2_^-^, NO_2_^-^, and NH_4_^+^ in filtered solutions were determined colorimetrically with sulfanilamide (with VCl_3_ for NO_3_^-^+NO_2_^-^, without VCl_3_ for NO_2_^-^) and salicylate/nitroprusside (for NH_4_^+^). The method used for NO_3_^-^+NO_2_^-^ determination utilizes VCl_3_, sulfanilamide and N-1-napthylethylenediamine under acidic conditions, which prevents Fe^2+^ interference in the determination of NO_3_^-^ concentrations (SI for additional discussion). Total dissolved nitrogen (TDN) was determined by measuring total NO_3_^-^ in solutions after digestion with potassium persulfate. The difference between TDN and the sum of NO_3_^-^+NO_2_^-^ and NH_4_^+^ is defined as dissolved organic nitrogen (DON). The concentration of ferrous iron (Fe(II)) in the LC was determined by mixing 2 ml of the LC suspension with 2 ml of 1 M HCl in an anaerobic chamber for 24 h. The mixture was filtered (0.2 μm membrane) and the filtrate analyzed for total Fe(II) with the Ferrozine reagent. Measured Fe(II) is defined as 0.5 M extractable Fe(II) in the system.

The ^15^N isotope ratio (^15^N/(^14^N+^15^N)) in NO_3_^-^ and NH_4_^+^ was determined. For NO_3_^-^, solutions were treated with a microbial denitrifier and the ^15^N isotope ratio in the resultant N_2_O gas was measured while dissolved NH_4_^+^ was trapped onto acidified discs [48]. Solutions were also freeze-dried and the solid residue reconstituted in 50 μL of deionized H_2_O. An aliquot (7 μL) of the reconstituted solutions was deposited onto an acidified disc for ^15^N isotope ratio determination of TDN. The magnitude of N immobilization (i.e., transformation of N_i_ to SON) is defined as the amount of ^15^N recovered in the solid-phase, calculated as the difference in ^15^N enrichment of the LC before and after incubation. Standard isotope mixing models [49] were used to calculate the fraction of tracer ^15^NO_3_^-^ in solid-phase, NO_3_^-^, NH_4_^+^, and DON pools. Nitrogen (^15^N) isotope ratio measurements were conducted at the Stable Isotope Facility at the University of California, Davis and at the Boston University Stable Isotope Laboratory.

## Results

### Nitrate, nitrite, and Fe(II) concentrations

**Fig 1:**
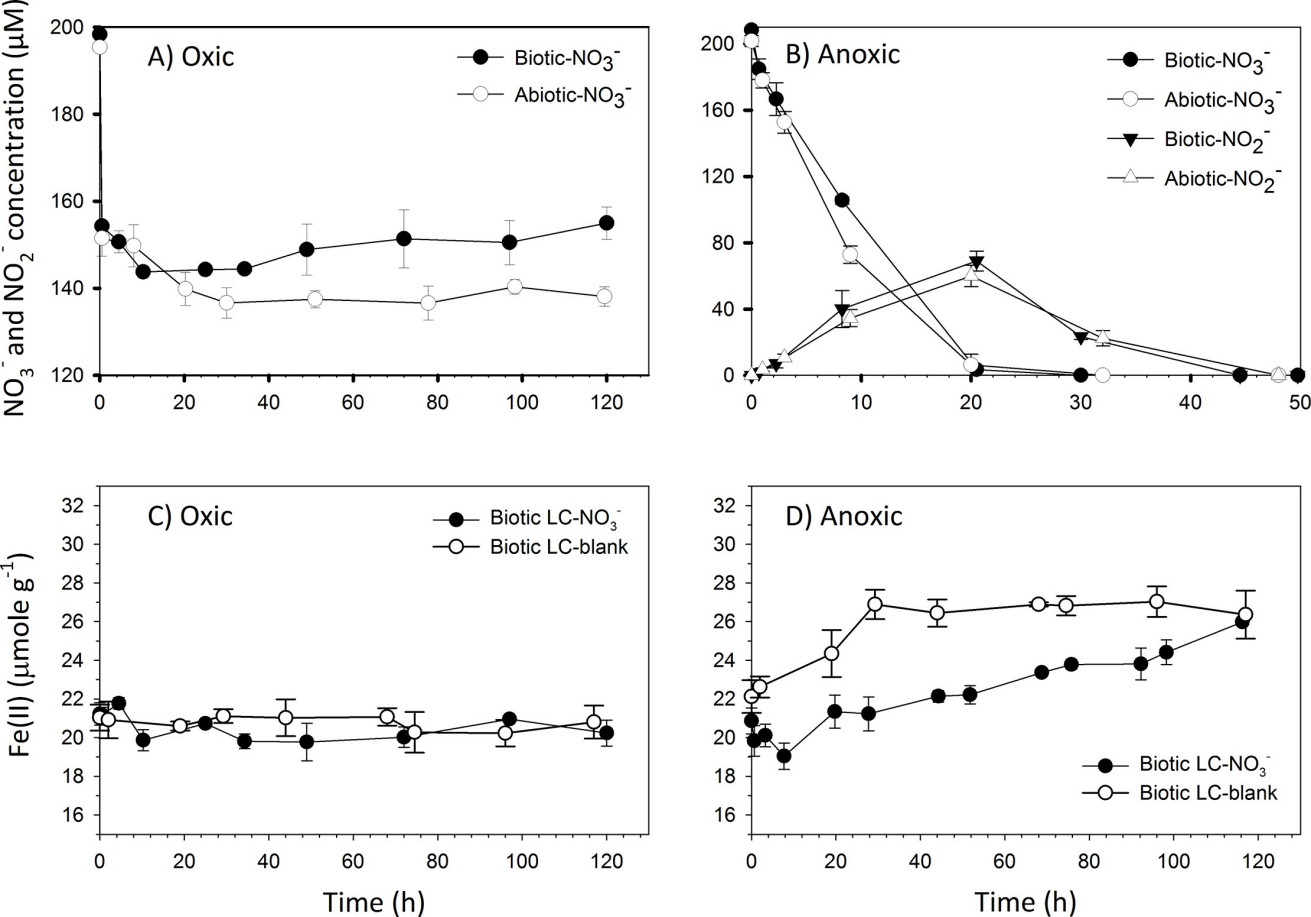
Nitrate (NO3-), nitrite (NO2-) and ferrous iron (Fe(II)) concentrations as a function of incubation time. Panels A and B represent NO_3_^-^ and NO_2_^-^ concentrations in experimental (NO_3_^-^ spiked) incubations under oxic and anoxic conditions, respectively. Panel C and D present 0.5 M HCl extractable Fe(II) concentrations in blank (no NO_3_^-^ spiked) and experimental (NO_3_^-^ spiked) incubations under biotic-oxic and biotic-anoxic conditions, respectively. Initial NO_3_^-^ concentration was 200 μM. Error bars represent standard deviation of duplicate values. Note different scale for x-axis in panel B.

Under oxic conditions, both the experimental biotic-oxic and abiotic-oxic (i.e., γ-irradiated oxic) systems illustrated initially abrupt, yet incomplete, decreases in aqueous NO_3_^-^ concentration (≈20%) immediately after its addition (**Fig 1A**). Similar initial (immediate) decreases were not observed in anoxic incubations (**Fig 1B**). These results therefore suggest that under oxic conditions a portion of the spiked NO_3_^-^ was rapidly transformed to other N species in abiotic-oxic incubations. Following an additional ≈5% decrease within 20 h, NO_3_^-^ concentration remained unchanged throughout the abiotic-oxic incubation but increased slightly towards the end of the biotic-oxic incubation (**Fig 1A**). A decrease in ^15^N isotope ratio in NO_3_^-^ (from 98 to 94 atom %) was found in solution at the end of the biotic-oxic incubation (**Table A in S1 File**), thus confirming that newly generated NO_3_^-^ contributed to the NO_3_^-^ pool, likely due to microbial nitrification. The ^15^N isotope ratio in NO_3_^-^ did not change in the abiotic-oxic incubation, indicating that there was no NO_3_^-^ production in γ-irradiated incubations.

Nitrate concentrations dropped to zero after ~20 h in anoxic incubations **(Fig 1B**). The highest NO_2_^-^ concentrations were also observed in these systems at ~ 20 h, and dropped to zero at ~50 h (**Fig 1B**). Nitrite detection in biotic-anoxic and abiotic-anoxic incubations indicates NO_3_^-^ went through reduction and that NO_3_^-^ reduction in the biotic-anoxic system was likely a chemical process since the patterns of change in NO_3_^-^ and NO_2_^-^ concentrations observed in the biotic-anoxic incubation are identical to those observed in the abiotic-anoxic incubation. It is worth noting that the amount of NO_2_^-^ at its peak concentration accounts for ~1/3 of the initial NO_3_^-^ spike. Under anoxic conditions, several processes may have led to NO_3_^-^/NO_2_^-^ disappearance: 1) NO_3_^-^ reduction to NH_4_^+^; 2) NO_3_^-^ reduction to NO_2_^-^ and then to gaseous nitrogen species (i.e., NO_x_, N_2_), which are lost to the atmosphere; 3) NO_3_^-^/NO_2_^-^ incorporation into either solid or dissolved organic matter (i.e., SON, DON). It is likely a combination of these processes was operative in both the biotic-anoxic and abiotic-anoxic incubations, as shown by the ^15^N isotope ratio data presented in **Table A in S1 File**.

The concentration of Fe(II) remained relatively constant throughout experimental and blank biotic-oxic incubations, indicating the addition of NO_3_^-^ had no influence on Fe(II) concentrations (**Fig 1C**). Fe(II) concentrations in blank biotic-anoxic incubations increased initially but remained constant after ~30 h (**Fig 1D**). In contrast, a decrease in Fe(II) concentrations was observed within 8 h in experimental biotic-anoxic incubations followed by a steady increase that resulted in equal concentration in blank and experimental incubations at 120h.

### Ammonium and dissolved organic nitrogen

**Fig 2:**
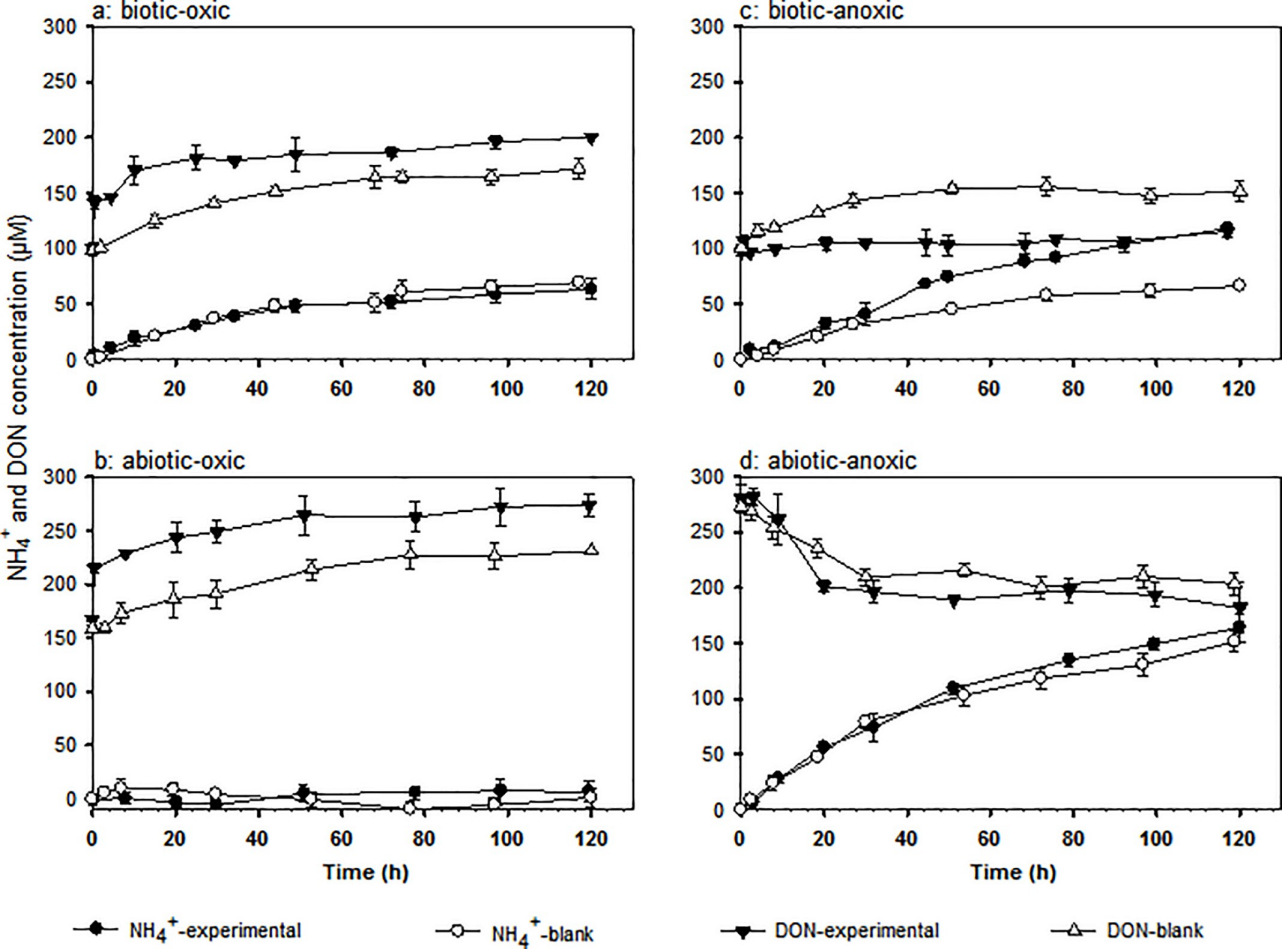
Ammonium (NH4+) and dissolved organic nitrogen (DON) concentrations in experimental (NO3- spiked) and blank (no NO3- spiked) incubations under four sets of conditions. Error bars represent standard deviation of duplicate values.

Experimental and blank incubations displayed significant changes in DON and NH_4_^+^ concentrations with time (**Fig 2**). DON concentrations in experimental biotic-oxic and abiotic-oxic incubations rose by ~40 μM immediately after NO_3_^-^ addition (**Fig 2A and 2B**), an increase equal in magnitude to the initial rapid decrease in NO_3_^-^ concentrations under oxic conditions. An increase in ^15^N enrichment was found in the DON pool in both the experimental biotic-oxic and abiotic-oxic system (**Table A in S1 File**). Collectively, these data confirm that ~17% of the ^15^N-NO_3_^-^ added was rapidly transformed to ^15^N-DON in γ-irradiated LC under oxic conditions. Following the initial rapid increase, DON in the experimental biotic-oxic and abiotic-oxic incubations rose gradually with time and paralleled DON increases in blank incubations. The same amount of NH_4_^+^ was produced in the experimental and blank biotic-oxic incubations (**Fig 2A**). Ammonium was at or close to the detection limit in experimental and blank abiotic-oxic incubations (**Fig 2B**).

Under biotic-anoxic conditions, DON concentrations in the blank incubation increased during the first ~50 h when a plateau was reached but remained constant in the experimental incubation (**Fig 2C**). Ammonium increased in both the blank and experimental biotic-anoxic incubations; but the NH_4_^+^ increase in the experimental system was twice that in the blank at the end of incubation (**Fig 2C**). In contrast, the blank and experimental abiotic-anoxic incubations showed a decrease in DON concentration and the greatest increase in NH_4_^+^ concentration among all systems **(Fig 2D**). The magnitude of NH_4_^+^ increase and DON decrease in the experimental abiotic-anoxic incubation are comparable to those in its blank incubation.

### Nitrogen immobilization

**Fig 3:**
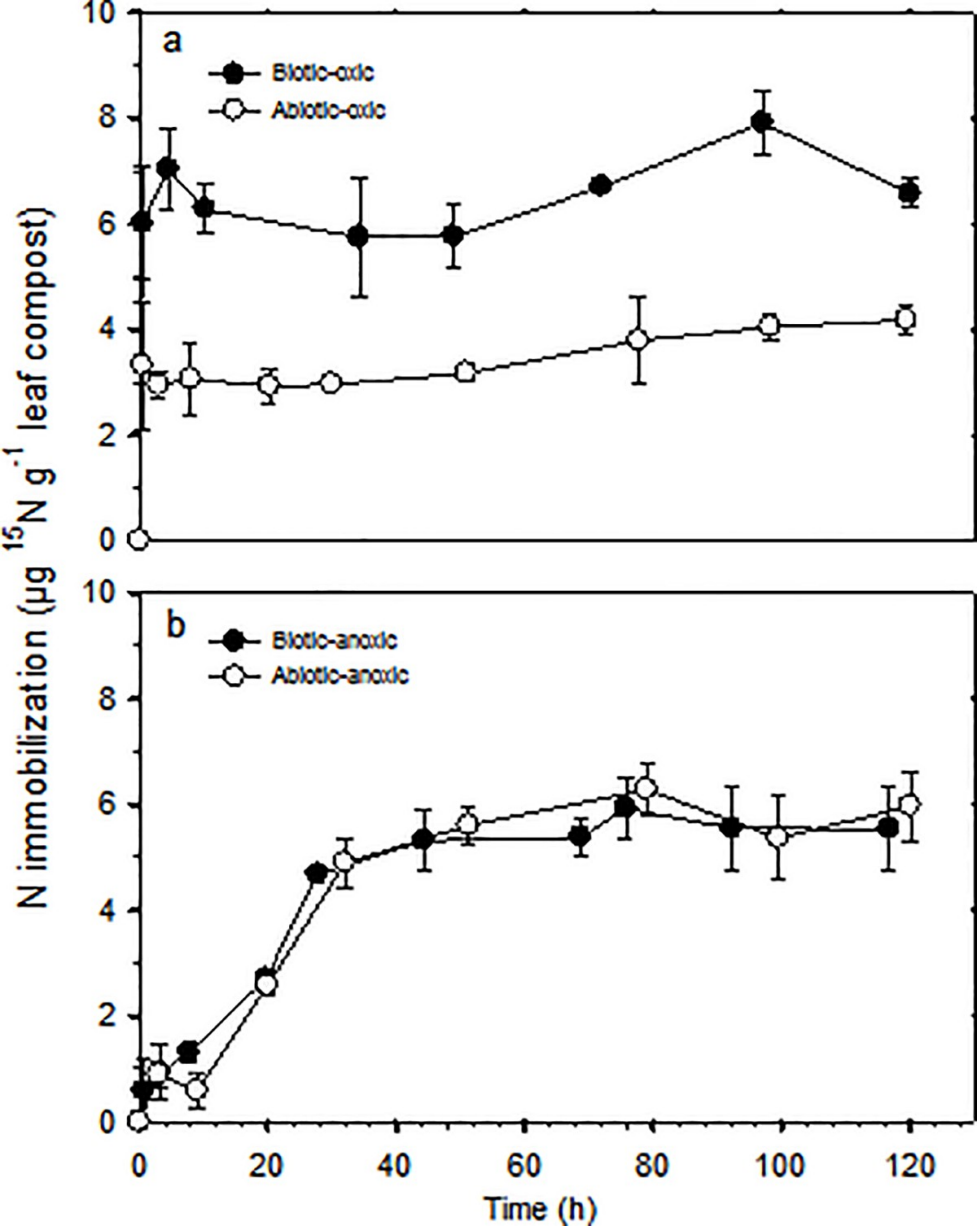
**Nitrogen immobilization in biotic and abiotic systems under oxic (a) and anoxic (b) conditions.** Error bars represent standard deviation of duplicate values.

Nitrogen immobilization in solid-phase leaf compost (**Fig 3**) fluctuated with time in the biotic-oxic incubation with an average of 6.2 μg ^15^N per g leaf compost. In the abiotic-oxic incubation, N immobilization increased slightly over time, with ~80% of the immobilization completed within 30 min of incubation. Thus, N was immobilized rapidly by organic matter under oxic conditions. If N immobilization in the biotic-oxic system is considered a combined result of biotic and abiotic processes, then the difference in magnitude between N immobilization in the biotic-oxic system and that in the abiotic-oxic system can be regarded as the magnitude of biotic N immobilization. As derived from the data shown in **Fig 3A**, under oxic conditions, ~66% of the overall N immobilization can be attributed to abiotic processes.

In contrast to oxic incubations, N immobilization proceeded gradually in the biotic-anoxic and abiotic-anoxic incubations (**Fig 3B**). N immobilization reached a plateau of ~6.0 μg ^15^N per g leaf compost at ~60 h, the time when NO_3_^-^ and NO_2_^-^ concentrations had dropped to zero. The similarity in NO_3_^-^ reduction dynamics (**Fig 1B**) and N immobilization (**Fig 3B**) between the biotic-anoxic and abiotic-anoxic systems suggest abiotic processes dominate NO_3_^-^ transformation under anoxic condition with 100% of the overall N immobilization attributed to abiotic processes. About 0.8% of the added ^15^N-NO_3_^-^ was retained in solid leaf compost in the experimental biotic-anoxic and abiotic-anoxic systems, and NO_3_^-^ was not converted to DON as evidenced by ^15^N isotope measurements (**Table A in S1 File**). By mass balance, it can be concluded that the majority of the spiked ^15^N-NO_3_^-^ was either reduced to gaseous N (lost from the system) or to NH_4_^+^ (remained in the system) under anoxic conditions.

We attempted to identify N species resulting from N immobilization processes using CP MAS ^15^N-NMR (**Figure A in S1 File and Tables B and C in S1 File**). Although the results are somewhat inconclusive (signal to noise ratios equal to or greater than 3:1 are desirable), they suggest new ^15^N (from ^15^N-NO_3_^-^ addition) was immobilized in the LC (i.e., a signal was present in experimental incubations while no signal was detected in blank incubations). ^15^N-NMR spectra suggest the formation of amine-N under oxic conditions whereas under anoxic conditions the dominant N species seem to be amide/aminoquinones and nitro/oxime [(R-NO_2_)/(R^1^(R/H)^2^C = NOH)] (see discussion in **S1 File**). These results suggest additional studies on the formation of organic N species, perhaps utilizing more challenging NMR experiments, are warranted.

## Discussion

### Nitrate transformation pathways

**Fig 4:**
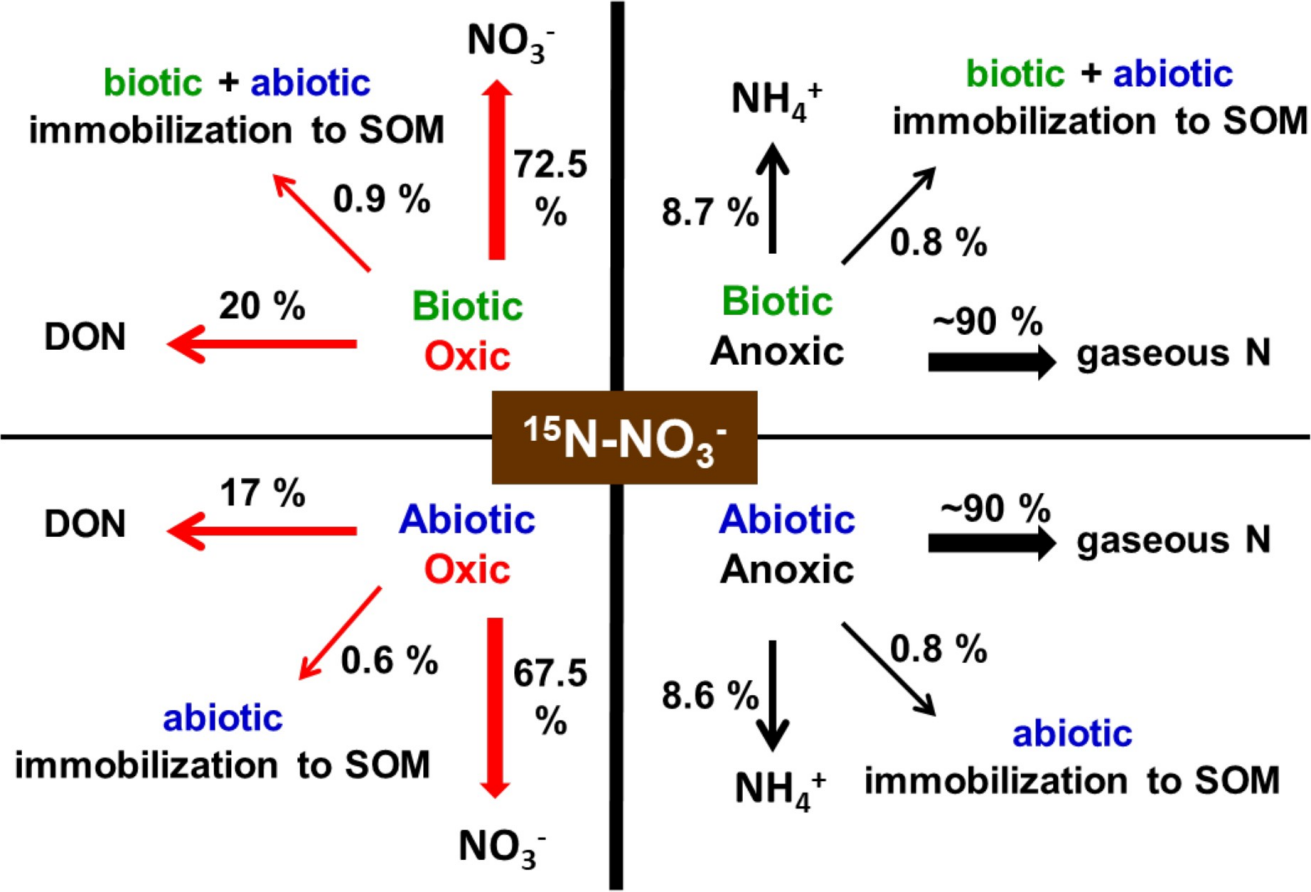
Schematic representation of ^15^N-NO3- transformation pathways as affected by incubation conditions. Percentage values indicate the amount of the initial ^15^N-NO_3_^-^ added (750 μg ^15^N / g LC) that partitioned into specific N species. Note: since no change in ^15^N was detected in the NH_4_^+^ pool of oxic incubations (**Table A in S1 File**), it is possible the remaining N label (~6.6% in biotic-oxic and ~14.9% in abiotic-oxic incubations) could have transformed to gaseous N species under oxic conditions [1,2].

Transformation pathways for NO_3_^-^ under the four incubation conditions are presented schematically in **Fig 4**. Our results indicate both redox and abiotic conditions govern the dynamics of NO_3_^-^ transformations in organic matter systems. Specifically, oxic incubations indicate that ^15^NO_3_^-^ was not recovered in the NH_4_^+^ pool; the dominant pathway for NO_3_^-^ transformation is conversion to DON, accounting for 20 and 17% of the spiked ^15^NO_3_^-^ in biotic-oxic and γ-irradiated abiotic-oxic systems, respectively (**Table A in S1 File**, **Fig 4**). Hence, under oxic conditions NO_3_^-^ is more prone to being incorporated abiotically into DOM than into SOM when incubations were conducted in suspension. A similar trend was also found in several studies [15,20,34]. Davidson et al. (2003) [25] proposed that NO_3_^-^ is first reduced to NO_2_^-^ prior to its incorporation into DOM. Their hypothesis is based on the results of two discrete experiments. The first experiment showed nitrate reduction by Fe(II) in the presence of a Cu catalyst under *anoxic* conditions; the second experiment showed nitrite incorporation into dissolved organic matter (i.e., solutions of several organic compounds). In the first experiment, a decrease in nitrate concentration with time, not an increase in nitrite concentration, was measured; therefore, it was not demonstrated that NO_2_^-^ is the intermediary between NO_3_^-^ and DOM. In addition, Schmidt and Matzner (2009) [45] showed that NO_2_^-^ transformation to DOM under *oxic* conditions did not occur after NO_2_^-^ was added to sterilized DOM. Matus et al. (2019) [23] however demonstrated that under abiotic-anoxic conditions, and in the presence of Fe^2+^, DOM reacts with NO_3_^-^ to form DON. Based on our experimental results we cannot confirm nor completely refute whether NO_3_^-^ was reduced to NO_2_^-^ under the oxic conditions in which NO_3_^-^ was converted to DOM (NO_2_^-^ was not detected in oxic incubations at values above 1 μM). However, we can use the Nernst equation to calculate the theoretical concentration of nitrite (NO_2_^-^) that would result from the NO_3_^-^/NO_2_^-^ redox couple: ½ NO_3_^-^ + H^+^ + e^-^ → ½ NO_2_^-^ + ½ H_2_O (E_h_^o^ = 0.834 V). With parameter values of pH = 6.5, E_h_ = 500 mV and [NO_3_^-^] = 200 μM (i.e., NO_3_^-^ initially added), we calculated [NO_2_^-^] = 4.1 μM, which is above our detection limit of 1 μM. Higher NO_2_^-^ concentrations are expected at lower redox potentials whereas lower NO_2_^-^ concentrations are expected at higher redox potentials. Our oxic incubations, with a redox potential of ~300 mV, should have in theory resulted in a nitrite concentration of 199 μM, a concentration easily detectable. Therefore, the conversion of NO_3_^-^ to DOM that we observed in experiments conducted under oxic conditions does not seem to follow the path hypothesized by Davidson et al. (2003) [25]. Although NO_2_^-^ was not detected, it is possible the kinetics of NO_2_^-^ consumption were as fast (or faster) than those of NO_2_^-^ production, which would have prevented NO_2_^-^ accumulation in the system. A potential pathway is that NO_3_^-^ undergoes an electrophilic aromatic substitution (aromatic nitration) in which a nitro group (R-NO_2_) is introduced into an organic chemical compound. Yet, we do not have conclusive evidence in support of this reaction as a legitimate possibility. Recent studies have demonstrated that UV irradiation effects the incorporation of nitrate and nitrite into natural organic matter via nitration and nitrosation yielding a variety of organic-N functionalities [50]. These reactions present additional evidence in support of abiotic pathways leading to the incorporation of inorganic N species into organic matter [51].

Under anoxic conditions, all of the spiked ^15^N-NO_3_^-^ was transformed within 24 h (**Fig 1B**). About 8.6% of the spiked ^15^N-NO_3_^-^ was recovered as ^15^N-NH_4_^+^ (**Table A in S1 File**, **Fig 4**) by the end of the biotic-anoxic and γ-irradiated abiotic-anoxic system incubations, with no ^15^N recovery in the DOM pool. Given that ~0.8% of ^15^N-NO_3_^-^ was immobilized into SOM and that relatively large concentrations of NO_2_^-^ were detected in anoxic incubations, we infer ~90% of the spiked ^15^N-NO_3_^-^ was reduced to gaseous N species. Our inference is supported by the work of Zhang et al. (2010) [34] in which the authors found that up to 51% of the spiked NO_3_^-^ was converted to N_2_ gas when NO_3_^-^ was incubated with forest soils anaerobically. Although gaseous N species were not measured in this study, the low E_h_ (-330 mV) in all anoxic incubations suggests a very low oxygen level conducive to N_2_ gas production.

Although Fe(II) is expected to play a role in NO_3_^-^ reduction, the results from biotic-oxic incubations show the addition of NO_3_^-^ had no influence on Fe(II) concentrations (**Fig 1C**), therefore indicating Fe(II) did not act as an electron source in biotic-oxic incubations (but perhaps as a catalyst). However, the addition of nitrate under anoxic conditions affected Fe(II) concentrations. Fe(II) concentrations were consistently lower in experimental compared to blank anoxic incubations (except at 120 h) suggesting Fe(II) was directly involved in NO_3_^-^ reduction (**Fig 1D**). These findings lend support for Davidson’s hypothesis that postulates NO_3_^-^ reduction by Fe^2+^ under anoxic conditions [25]. Potential electron sources that would support NO_3_^-^ reduction under anoxic conditions are reducing organic functional groups and Fe(II) present in the leaf compost (Fe(II) = 1.2 g Fe(II)/kg = 21.5 μmole Fe(II)/g = 43 μmole Fe(II) in reaction vessel; total Fe = 4.8 g Fe/kg = 85.9 μmole Fe/g = 171.8 μmole Fe in reaction vessel). Since 100 μmole NO_3_^-^ were added to each experimental incubation, and given the fact that all of the added NO_3_^-^ was transformed (reduced) to other species under anoxic conditions, reduction of NO_3_^-^ to NO_2_^-^ alone would require 100 μmole of electrons. Using the concentration of NO_3_^-^ (~100 μM in solution), NO_2_^-^ (~40 μM in solution) and Fe(II) (~4 μmole Fe(II)/g = difference between blank and experimental values) at 8 h in biotic-anoxic incubations, and our experimental parameters (2 g LC in 500 ml suspension), we calculate that ~50 μmole of NO_3_^-^ were consumed, ~20 μmole of NO_2_^-^ were produced and that Fe(II) was reduced by ~8 μmole in experimental compared to blank incubations. These calculations indicate Fe(II) provided electrons for NO_3_^-^ reduction but was not the sole electron source for NO_3_^-^ reduction in anoxic incubations, otherwise the Fe(II) concentration would have decreased considerably more. If Fe(II) had served as a catalyst (i.e., a substance that increases the rate of a chemical reaction without itself undergoing any permanent chemical change), the reduction of NO_3_^-^ to NO_2_^-^ would take place but the concentration of Fe(II) would remain constant. We suggest organic matter (i.e., LC in this work) provided electrons for NO_3_^-^ reduction. Some studies have found that the rate of denitrification is highly correlated to DOC concentration in groundwater [52,53], suggesting microorganisms may prefer DOC over solid organic C (SOM) as the electron source to fuel denitrification. In this study, experimental and blank DOC concentrations in biotic-anoxic systems increased with time, independent of nitrate addition (**Figure B in S1 File**), thus suggesting the LC may have served as an additional electron source for NO_3_^-^ reduction in anoxic incubations.

### Nitrogen source for ammonium production

^15^N-NO_3_^-^ was not recovered in the NH_4_^+^ pool of the experimental oxic systems which implies ^15^N-NO_3_^-^ was not directly or indirectly converted to NH_4_^+^. The contrast in NH_4_^+^ production between the biotic-oxic and γ-irradiated abiotic-oxic systems (**Fig 2A and 2B**) indicate microbial activity was required for NH_4_^+^ production. Production of NH_4_^+^ can be explained by classic theory, namely, microbial generation of low molecular weight organics from solid-phase OM using extracellular enzymes, and organic N assimilation followed by NH_4_^+^ excretion [54,55]. The fact that NH_4_^+^ was not labeled but DON in the experimental biotic-oxic system was highly enriched in ^15^N due to ^15^N-NO_3_^-^ conversion to DON is striking (**Table A in S1 File**). Although microbial utilization of non-labeled DON might be coincidental, we speculate microbes could release enzymes that convert solid phase OM into NH_4_^+^ (e.g., enzymes that cleave amino groups from proteinaceous material). The addition of NO_3_^-^ did not seem to influence the outcome of microbial NH_4_^+^ production, as evidenced from the fact that the same amount of NH_4_^+^ was produced in experimental and blank oxic incubations. Schmidt et al. (2011) [56] also found that NO_3_^-^ addition exerted no impact on the rate of DOM microbial mineralization.

As mentioned above, NH_4_^+^ produced in the experimental biotic-anoxic incubation was twice that in the blank (**Fig 2C**). This additional NH_4_^+^ production can be attributed to two processes, one of which is ^15^N-NO_3_^-^ reduction to ^15^N-NH_4_^+^ as confirmed by increased ^15^N enrichment in the NH_4_^+^ pool (**Table A in S1 File**). We cannot confirm, however, whether the reduction was a biotic or abiotic process. Our calculation indicates that ~8.7% of the spiked ^15^N-NO_3_^-^ was reduced to ^15^N-NH_4_^+^ (**Table A in S1 File**, **Fig 4**), accounting for ~14% of the total NH_4_^+^ production. Besides differences in NH_4_^+^ production, the experimental and blank biotic-anoxic systems also differ in DON production: DON concentration remained unchanged in the experimental biotic-anoxic incubation but increased in the blank. This suggests DON was mineralized (reduced) to NH_4_^+^ in the experimental biotic-anoxic system but not in the blank. A potential explanation for such difference is that DON mineralization under anoxic conditions was a microbially-driven process enhanced in the experimental incubation due to NO_3_^-^ addition. ^15^N-NO_3_^-^ was also reduced to ^15^N-NH_4_^+^ (~8.6%) in the experimental γ-irradiated abiotic-anoxic system (**Table A in S1 File**, **Fig 4**), accounting for ~10% of the total NH_4_^+^ production (**Fig 2D**). The remaining NH_4_^+^ production in experimental as well as blank γ-irradiated abiotic-anoxic incubations should be attributed to organic nitrogen chemical reduction. In these γ-irradiated anoxic systems, since a decrease in DON was associated with an increase in NH_4_^+^ production, it is most reasonable to conclude that DON was chemically reduced to NH_4_^+^ under abiotic-anoxic conditions.

### Magnitude of nitrogen immobilization within SOM

The magnitude of N immobilization within SOM ranged from 4.1 to 6.6 μg ^15^N per g LC, accounting for 0.6–0.9% of the total added ^15^N. Nitrogen immobilization, expressed as percentage, appears to be lower than figures reported in similar studies. Dail et al. (2001) [15] and Fitzhugh et al. (2003) [20] reported that 5–10% of the total added ^15^N-NO_3_^-^ (4–5 μg ^15^N per g soil) was immobilized by live or sterilized O-horizon forest soils; such immobilization translates to a magnitude less than 1 μg ^15^N per g soil. Thus, our seemingly low % N immobilization by leaf compost can simply be explained by the larger total ^15^N-NO_3_^-^ input. Moreover, using a N immobilization value of 6.5 μg N g^-1^ leaf compost and a density of 1 g cm^-3^, we calculate 5.2 kg N ha^-1^ would be immobilized (stored) within an organic matter layer 8 cm in thickness (e.g., O-horizon of a forest soil). The amount of N stored within SOM would be 3.2 kg N ha^-1^ using an N immobilization value of 4 μg N g^-1^ leaf compost. Our estimates of N immobilization help to explain, at least in part, the observed decline in N export in some forest ecosystems [57].

As NO_3_^-^ input from fossil fuel combustion and fertilizer application continues to bypass the natural N cycle, changes in climatic conditions (e.g., frequency and intensity of precipitation that affects redox gradients and microbial growth rates and metabolism) might enhance the prevalence of abiotic transformations (i.e., chemical processes) that shift pathways and N species concentrations from those controlled by biota.

## Supporting information

S1 File **(Table A) Changes in** ^**15**^**N atom % in four N pools after 5-day incubation under a factorial of biotic/abiotic and oxic/anoxic conditions. (Table B) Compilation of** ^**15**^**N solid-state NMR data collection parameters used by referenced publications and in the current study. (Table C) Peak assignment for signals of solid-state CP/MAS** ^**15**^**N NMR. (Figure A) Solid-state CP MAS 15N NMR spectra of leaf compost after incubation with** ^**15**^**N labeled NO**_**3**_^**-**^ **in Oxic (a) and Anoxic (b) systems.** The spectra of the blank (rinsed, no NO_3_^-^ addition) leaf compost (c, e) and of the original (non-rinsed, no NO_3_^-^ addition) leaf compost (d, f) are also shown. Contact times (CT) of 2 ms and 5 ms were used in data collection as indicated in each panel. **(Figure B) Nitrate (NO**_**3**_^**-**^**) and dissolved organic carbon (DOC) concentrations in experimental (NO**_**3**_^**-**^ **spiked) and blank incubations under biotic-anoxic (a) and abiotic-anoxic (b) conditions.** Initial NO_3_^-^ concentration was 200 μM. Error bars represent standard deviation of duplicate values. Note left y-axis pertains to NO_3_^-^ data and right y-axis pertains to DOC data. **(Figure C) Measured NO**_**3**_^**-**^ **concentration (y-axis) in solutions containing 10, 25, 80 and 200** μ**M NO**_**3**_^**-**^ **and each containing 0, 1, 5, 10, 50, 100, 400 and 800** μ**M Fe**^**2+**^. The 1:1 actual:measured NO_3_^-^ concentration is represented by the solid line. Symbols represent all data points (average of 3 experimental replicates) for 200 (black circles), 80 (red squares), 25 (blue triangles) and 10 (pink diamonds) μM NO_3_^-^ concentrations. **(Figure D) Measured NO**_**3**_^**-**^ **concentration in solutions containing 10, 25, 80 and 200** μ**M NO**_**3**_^**-**^ **in the presence of 0, 1, 5, 10, 50, 100, 400 and 800** μ**M Fe**^**2+**^. Dashed horizontal lines represent actual NO_3_^-^ concentrations. Symbols represent measured NO_3_^-^ concentrations (average of three experimental replicates) and error bars their standard deviation (200, circles; 80, squares; 25, triangles; 10, diamonds). (A) shows all of the data; for clarity (B) presents an expanded x-axis with results for 0–10 μM Fe^2+^. **(Figure E) Sequence of reactions involved in the analytical method used for the determination of NO**_**3**_^**-**^ **concentrations.**
(DOCX)
