# Supplementary material for: Nitrate transformation and immobilization in particulate organic matter incubations: Influence of redox, iron and (a)biotic conditions
Source: PLoS One. 2019 Jul 5;14(7):e0218752. doi: 10.1371/journal.pone.0218752 (PMC6611582; doi:10.1371/journal.pone.0218752)
Supplement: S1 File — (Table A) Changes in 15N atom % in four N pools after 5-day incubation under a factorial of biotic/abiotic and oxic/anoxic conditions. (Table B) Compilation of 15N solid-state NMR data collection parameters used by referenced publications and in the current study. (Table C) Peak assignment for signals of solid-state CP/MAS 15N NMR. (Figure A) Solid-state CP MAS 15N NMR spectra of leaf compost after incubation with 15N labeled NO3- in Oxic (a) and Anoxic (b) systems. The spectra of the blank (rinsed, no NO3- addition) leaf compost (c, e) and of the original (non-rinsed, no NO3- addition) leaf compost (d, f) are also shown. Contact times (CT) of 2 ms and 5 ms were used in data collection as indicated in each panel. (Figure B) Nitrate (NO3-) and dissolved organic carbon (DOC) concentrations in experimental (NO3- spiked) and blank incubations under biotic-anoxic (a) and abiotic-anoxic (b) conditions. Initial NO3- concentration was 200 μM. Error bars represent standard deviation of duplicate values. Note left y-axis pertains to NO3- data and right y-axis pertains to DOC data. (Figure C) Measured NO3- concentration (y-axis) in solutions containing 10, 25, 80 and 200 μM NO3- and each containing 0, 1, 5, 10, 50, 100, 400 and 800 μM Fe2+. The 1:1 actual:measured NO3- concentration is represented by the solid line. Symbols represent all data points (average of 3 experimental replicates) for 200 (black circles), 80 (red squares), 25 (blue triangles) and 10 (pink diamonds) μM NO3- concentrations. (Figure D) Measured NO3- concentration in solutions containing 10, 25, 80 and 200 μM NO3- in the presence of 0, 1, 5, 10, 50, 100, 400 and 800 μM Fe2+. Dashed horizontal lines represent actual NO3- concentrations. Symbols represent measured NO3- concentrations (average of three experimental replicates) and error bars their standard deviation (200, circles; 80, squares; 25, triangles; 10, diamonds). (A) shows all of the data; for clarity (B) presents an expanded x-axis with results for [file pone.0218752.s001.docx]

**Supporting Information**

**Nitrate transformation and immobilization in particulate organic matter incubations: Influence of redox, iron and (a)biotic conditions**

Fiona R. Kizewski^2^, Jason P. Kaye^2^, and Carmen Enid Martínez^1^ *

^1^ Soil and Crop Sciences, School of Integrative Plant Science, Cornell University, Ithaca, NY 14853 USA

^2^ Department of Ecosystem Science and Management, The Pennsylvania State University, University Park, PA 16802 USA

^*^Corresponding author:

Carmen Enid Martínez [cem20@cornell.edu](mailto:cem20@cornell.edu)

Cornell University, Ithaca, NY 14853

607-255-0895 (office); 607-255-2644 (fax)

**Supporting Information**

Contains 13 pages including 3 tables, 5 figures and text, as follows:

I. Results from ^15^N isotope ratio determinations (Table A).

II. Nitrogen speciation by solid state ^15^N NMR spectroscopy (methods, results, discussion, Figure A, Table B, Table C).

III. Influence of gamma(γ)-irradiation on dissolved organic carbon (discussion, Figure B).

IV. Tests for Fe^2+^ interference in the determination of NO_3_^-^ concentrations (methods, Figures C, D, and E).

V. References.

**I. Results from ^15^N isotope ratio determinations.**

**Table A**. Changes in ^15^N atom % in four N pools after 5-day incubation under a factorial of biotic/abiotic and oxic/anoxic conditions.

|  | **NH_4_^+^** | **NO_3_^-^** | **DON** | **SON** |
| --- | --- | --- | --- | --- |
| Oxic (biotic-oxic) | no change**^1^** | 98%**^2^** → 94% | na**^4^** → 20% | na → 0.405%**^5^** |
| γ-Oxic (abiotic-oxic) | no change**^1^** | no change**^3^** | na → 17% | na → 0.388% |
| Anox (biotic-anoxic) | na→ 8.75% | not measured | no change**^1^** | na → 0.399% |
| γ-Anox (abiotic-anoxic) | na→ 8.56% | not measured | no change**^1^** | na → 0.398% |

1. “no change” denotes ^15^N enrichment in NH_4_^+^ and DON did not change from natural abundance (0.367%).
2. 98% is the enrichment level of the K^15^NO_3_ used to prepare nitrate incubation solutions.
3. “no change” denotes ^15^N enrichment in NO_3_^-^ did not change from 98% (^15^N abundance in K^15^NO_3_).
4. “na” = natural abundance of ^15^N (0.367%)
5. Standard isotope mixing models (Fry, 2006) were used to calculate the fraction of tracer ^15^NO_3_^-^ in solid-phase (leaf compost, LC).

atom % _sample_*M_sample_ = atom % _source1_*M_source1_ + atom % _source2_*M_source2_

source1 = blank leaf compost

source 2 = tracer ^15^NO_3_^-^

sample = incubated leaf compost

M = mass; M_sample_ = M_source1_ + M_source2_

**II. Nitrogen speciation by Solid State ^15^N NMR Spectroscopy.**

***Materials and Methods.*** Nitrogen species present in the LC were identified by solid-state cross polarization (CP) magic angle spinning (MAS) ^15^N NMR spectroscopy. NMR experiments were conducted at the NMR facility of The Pennsylvania State University using a Bruker AV-300 and a Chemagnetics/Varian Infinity 500 with a 4mm rotor. The line-broadening factor was 40Hz for the 300 MHz instrument and 50Hz for the 500 MHz instrument. Additional data collection parameters are listed in **Table B**. In order to select a contact time (CT) that would enhance non-amide N signals that might arise from NMR experiments, the original (non-rinsed, no NO_3_^-^ addition) and blank (rinsed, no NO_3_^-^ addition) leaf compost (designated hereafter as LC-original and LC-blank, respectively) were each run with 2 ms and 5 ms CT. A shorter CT (e.g., 2 ms) favors the detection of H’s that are close to N (e.g., in amides) while longer CT (e.g., 5 ms) should in theory detect H’s that are far away from N (e.g., heterocyclic N) as well as H’s that are close to N. Longer CT however results in increased dephasing and therefore in a decrease of signal intensity. In addition, CP MAS **^15^**N-NMR spectra were collected for the LC incubated with ^15^NO_3_^-^ under Oxic and Anox conditions with 5 ms CT.

***Results****.* Peak assignment for signals of solid-state CP/MAS ^15^N NMR are presented in **Table C**. The spectrum of the LC-original (non-rinsed, no NO_3_^-^ addition) collected using 2 ms contact time (**Fig Af**) shows a prominent peak at ~120 ppm, attributable to amide-N. The same peak appears in the spectrum of LC-original collected using 5 ms contact time (**Fig Ad**), but as expected, the amide-N signal was greatly suppressed with a longer contact time. Comparison of the spectrum for LC-blank (rinsed, no NO_3_^-^ addition) collected with 2 ms contact time (**Fig Ae**) to that of the LC-original (**Fig Af**) indicates rinsing (water and KCl) removed a significant amount of amide-N. The latter suggests a fraction of amide-N originally present in the leaf compost was leached with water and salt. Most importantly, the spectrum of the LC-blank collected using 5 ms contact time shows essentially no identifiable peaks (**Fig Ac**), thus indicating the amide-N signal of the LC was effectively suppressed by rinsing and use of a longer contact time. The observed chemical shifts in incubated (experimental) leaf compost samples (**Figs Aa and Ab**) are therefore the result of ^15^N immobilization.

The CP MAS ^15^N-NMR spectra of the leaf compost incubated under Oxic and Anox conditions are shown in **Figs Aa and Ab**. Signals in the 0-60 ppm region represent the most reduced N, including ammonium and various amine groups such as those in amino acids or amino sugars (Thorn and Mikita, 2000). For the leaf compost incubated under Oxic conditions, amine-N contributes to the resonance in the 28-38 ppm range with its maximum located at 35 ppm. Chemical shifts from 100 to 150 ppm represent primary and secondary amides in the form of peptide-N and possibly N-acetyl N (Thorn and Cox, 2009). Thorn and Cox (2009) demonstrated that peak intensity at 120 ppm increased substantially after the Elliot soil humic acid was treated with ^15^N labeled glycine, a reaction that is expected to generate aminoquinones via Michael addition. Therefore, signals in the 100-150 ppm region of the spectrum for the Anox leaf compost are assigned to amide/aminoquinones species. Signals in the downfield region from 330 to 430 ppm represent the most oxidized N species. Chemical shifts from 330 to 390 ppm correspond to nitrate, nitro (R-NO_2_), and oxime N (Thorn and Cox, 2009, Thorn and Mikita, 1992, Thorn and Mikita, 2000). For the leaf compost incubated under Anox conditions, oxime and nitro N, represented by chemical shifts at 330-390 ppm, also seem to appear in the spectrum as a result of N immobilization processes.

***Discussion****.* Solid-state ^15^N NMR spectroscopy has been widely applied for the characterization of organic nitrogen in soils, soil clay fractions, plant material and organic matter (De la Rosa et al., 2013, Smernik and Baldock, 2005a, Smernik and Baldock, 2005b, Thorn and Cox, 2009, Thorn and Mikita, 1992, Thorn and Mikita, 2000). Data collection parameters used in several studies are listed in **Table B** and a compilation of peak assignments for signals in solid-state CP/MAS ^15^N-NMR spectra are presented in **Table C**. Although the studies shown in **Table B** differ in sample type, total N content, 15N enrichment levels and organic N species, the commonly used contact time of 1-2 ms has prevented detection of organic N species other than amide-N, even in highly enriched plant materials (Smernik and Baldock, 2005b). In contrast, a longer contact time (i.e., 5 ms) permitted the detection of organic N species in the incubated leaf compost samples, despite the fact that ^15^N enrichment was only slightly above natural abundance (**Fig A**). While immobilization of NO_3_^-^ is seldom considered in the context of reduction, the highest intensity amine-N peak at 35 ppm in **Fig Aa** signifies ^15^NO_3_^-^ underwent reduction through immobilization under oxic conditions. It is uncertain whether this reduction process was biotic or abiotic since only the ^15^N NMR spectra of leaf compost samples incubated under biotic conditions were collected. As reported by Thorn and Mikita (2000), ^15^N labeled nitrite was predominantly converted to oxime (R^1^(R/H)^2^C=NOH) and nitro (R-NO_2_), which gave rise to a strong resonance at around 370 ppm. In the experimental Anox incubation, a gradual N immobilization was accompanied by ^15^NO_3_^-^ reduction to NO_2_^-^. Additionally, N immobilization in the Anox system reached a plateau at 60 h when all NO_2_^-^ had disappeared from the system (**Fig 1b**). The signal at 371 ppm in the Anox leaf compost spectrum (although somewhat noisy) suggests that NO_2_^-^, a NO_3_^-^ reduction intermediate, was captured by the process of immobilization and transformed to oxime and nitro organic functionalities. The dominant species resulting from N immobilization in Oxic and Anox incubations differed, thus suggesting immobilization processes may occur through different reaction mechanisms depending on the makeup of the solution phase and on the reducing capacity of the solid phase.

**Table B**. Compilation of ^15^N solid-state NMR data collection parameters used by referenced publications and in the current study.

| **Sample features**  **/Parameters** | **Smernik and Baldock, 2005a** | **Smernik and Baldock, 2005b** | **Thorn and Cox, 2009** | **de la Rosa et al., 2013** | **Present investigation by Kizewski et al.** |
| --- | --- | --- | --- | --- | --- |
| **Sample** | soil clay fractions | wheat shoots and roots | Elliot soil fulvic and humic acid | soil incubated with K^15^NO_3_ for 28 days | rinsed LC incubated with K^15^NO_3_ for 5 days, and blank and original LC |
| **N content (wt %)** | 2.6-5.2% | 0.6-1.8% | 2.72-4.14% | 0.1% | 1.9% |
| **^15^N enrichment** | natural (0.367 atm %) | > 90 atm % | natural (0.367 atm %) | ~ 2.0 atm % | 0.4 atm % and natural (0.367 atm %) |
| **Instrument** | Varian Unity INOVA | Varian Unity INOVA | Chemagnetics CMX | Bruker DMZ and Varian INOVA | Bruker AV-300 and Chemagnetics/Varian Infinity 500 |
| **Magnetic field strength** | 400 MHz | 400 MHz | 200 MHz | 400 and 600 MHz | 300 MHz and 500 MHz |
| **Solid-state ^15^N-NMR technique** | CP MAS with high-power ^1^H decoupling | CP MAS | CP MAS | CP MAS | CP MAS |
| **Frequency** | 40.5 MHz | 40.5 MHz | 20.3 MHz | 40.54 MHz | 30.5 MHz |
| **Pulse delay** | 1 s | 2-4 s | 0.2-0.5 s | 0.15- 0.5s | 1 s |
| **Spinning speed** | 5 KHz | 5 KHz | 5 KHz | 5-8 kHz | 5 KHz |
| **Acquisition time** | 100 ms | 100 ms | 17.051 ms | N/A | 17.25 ms |
| **Number of scans** | 145,000-245,000 | 1,000-5,000 | 1,000,000-10,000,000 | 50,000-200,000 | 102,400 |
| **Contact time** | 1 ms | 1 ms | 2 ms | 1 ms | 2 ms and 5 ms |
| **Reference** | glycine at -347 ppm | glycine at -347 ppm | glycine at 32.6 ppm; ammonia 0.0 ppm | glycine at -347 ppm | glycine at 34.95 ppm |
| **Signal detected** | amide N | strong amide N; minor amino N | strong amide N; minor amino N | strong amide N; minor amino N | 5 ms CT: no distinct peak in blank; amine-N in oxic-biotic; amide/aminoquinone and nitro/oxime in anoxic-biotic |

**Figure A.** Solid-state CP MAS 15N NMR spectra of leaf compost after incubation with ^15^N labeled NO_3_^-^ in Oxic (a) and Anoxic (b) systems. The spectra of the blank (rinsed, no NO_3_^-^ addition) leaf compost (c, e) and of the original (non-rinsed, no NO_3_^-^ addition) leaf compost (d, f) are also shown. Contact times (CT) of 2 ms and 5 ms were used in data collection as indicated in each panel.


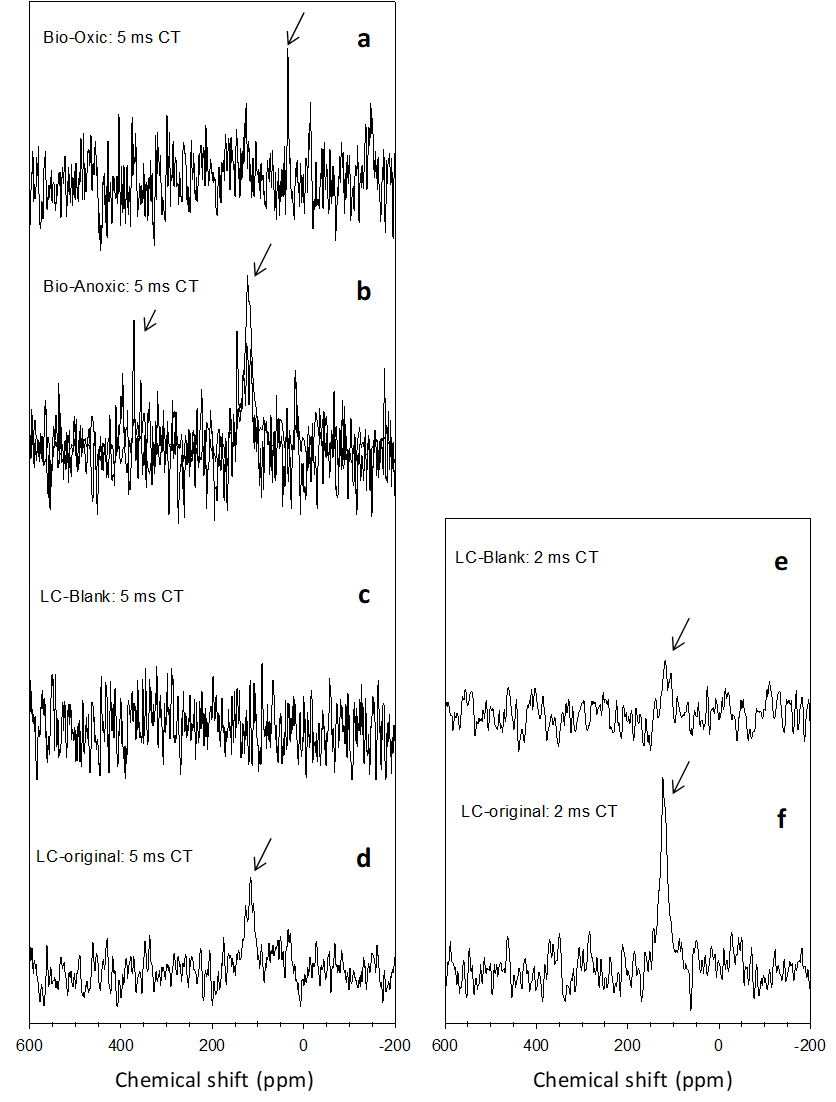


**Table C**. Peak assignment for signals of solid-state CP/MAS ^15^N NMR.

| **Chemical Shift (ppm)** | **Functional Groups** |
| --- | --- |
| 0-60 | ammonium, amines |
| 60-150 | 1° and 2° amides, N-acetyl N, aminoquinones |
| 150-280 | nitrile, hydroxamic acid, heterocyclic N |
| 280-330 | heterocyclic N |
| 330-390 | nitrate, nitro (R-NO_2_), oxime N |
| 390-430 | nitrosophenols (R-NO) |

**III. Influence of gamma**(**γ)-irradiation** **on dissolved organic C and N.**

Abiotic conditions (sample sterilization) were attained by gamma(γ)-irradiation of the leaf compost. Although γ-irradiation was used to achieve total microbial inactivation (i.e., loss of the ability to divide thus preventing subsequent microbial growth and the production of new enzyme molecules), enzyme activity in soil have shown variable sensitivity to irradiation. One drawback in soil enzymology studies is that most determinations of extracellular enzyme activity have been performed without separating microbial activity from enzymatic activity.

Initial (t=0) DOC concentrations in the abiotic-anoxic system (~65 mg C/L) were higher than in the biotic-anoxic system (~45 mg C/L) (**Fig B**). Similar trends in DOC concentration were however observed in both biotic and abiotic anoxic systems, with DOC in abiotic-anoxic incubations remaining higher (~92 mg C/L) than in biotic-anoxic incubations (~65 mg C/L) at the end of the experiment (t=120h). Note the addition of nitrate had no effect on DOC concentrations and trends as indicated by comparison of experimental and blank incubations. Similar impact on DOC was reported by Dail et al. (2001) who illustrated that DOC concentration in γ-irradiated O- and A- soil horizons was 10 and 15 times that of non-irradiated soils, respectively. Using UV-Vis and fluorescence spectroscopy, Berns et al. (2008) found that autoclave and γ-irradiation decreased organic carbon’s aromaticity and polymerization. In addition to higher initial DOC concentrations, higher DON concentrations were maintained during abiotic incubations (**Fig 2**). We also found more Fe(II) in the γ-irradiated leaf compost, suggesting γ-irradiation has caused some electron redistribution. These observations suggest chemical structures in organic matter might be altered to different extents by all sterilization techniques.

**Figure B**. Nitrate (NO_3_^-^) and dissolved organic carbon (DOC) concentrations in experimental (NO_3_^-^ spiked) and blank incubations under biotic-anoxic (a) and abiotic-anoxic (b) conditions. Initial NO_3_^-^ concentration was 200 µM. Error bars represent standard deviation of duplicate values. Note left y-axis pertains to NO_3_^-^ data and right y-axis pertains to DOC data.

**IV. Tests for Fe^2+^ interference in the determination of NO_3_^-^ concentrations.**

We tested the analytical method used in this study for potential interference of Fe^2+^ in the determination of NO_3_^-^ concentrations. It has been shown that Fe^2+^ interferes with NO_3_^-^ determination in methods that use high pH buffers (e.g., NH_4_Cl/EDTA) and/or a cadmium column (Colman et al., 2007), whereas other studies have reported the absence of Fe^2+^ interference in the quantification of NO_3_^-^ concentrations (Torres-Cañabate et al., 2008). We therefore tested our method for Fe^2+^ interference in NO_3_^-^ determination using a range of Fe^2+^ (0-800 µM, equal to 0-44.7 mg L^-1^) and NO_3_^-^ (10-200 µM, equal to 0.62-12.4 mg L^-1^) concentrations. As clearly shown by **Figs C-E**, and as discussed below, our measurements of NO_3_^-^ concentrations were not subjected to Fe^2+^ interference.

More specifically, we used a combination of four NO_3_^-^ concentrations (200, 80, 25 and 10 µM, equivalent to 12.4, 4.96, 1.55 and 0.62 mg L^-1^, respectively) and eight Fe^2+^ concentrations (800, 400, 100, 50, 10, 5, 1 and 0 µM, equivalent to 44.7, 22.3, 5.6, 2.8, 0.56, 0.28, 0.06 and 0 mg L^-1^, respectively) to test the analytical method used in our studies (Doane and Horwath, 2003). These nitrate and Fe^2+^ concentrations encompass the concentration range observed in our study and also include lower and higher Fe^2+^ concentrations. As clearly shown in **Figs C and D**, Fe^2+^ did not interfere in the determination of NO_3_^-^ concentrations, that is, the concentrations of NO_3_^-^ were not underestimated by the analytical method used in our studies. Clearly, Fe^2+^ did not interfere with the reduction process nor with color development or detection. It is important to note the differences between the protocol we used and protocols that use high pH buffers and/or phosphate filtration. First, we used VCl_3_ as the reducing agent for NO_3_^-^, and not a “Cd column” (i.e., a copper coated granulized cadmium reduction column). Second, we used a UV-Vis spectrophotometer in our analyses and not a flow-injection autoanalyzer (Lachat) which requires use of a Cd column for NO_3_^-^ reduction. Third, we did not use NH_4_Cl/EDTA, DTPA or imidazole because Fe^2+^ has been shown to sometimes interfere with NO_3_^-^ measurements when these pH 7.5-8.5 buffers are used with the analytical method. Our analytical method utilizes VCl_3_, sulfanilamide and N-1-napthylethylenediamine ***under acidic conditions*** (**Fig E**). It has been acknowledged (e.g., Yang et al., 2012) that a primary source of error resulting in underestimates of NO_3_^-^ concentrations is the age of the Cd column, that is, its efficacy in reducing NO_3_^-^ and not necessarily or solely Fe^2+^ interference. Among the advantages of the VCl_3_/sulfanilamide/N-1-napthylethylenediamine protocol is that the samples will turn yellow in the presence of NO_3_^-^, that is, if not all of the NO_3_^-^ is reduced to NO_2_^-^, thus preventing an underestimation of NO_3_^-^ concentration due to incomplete reduction.

**Figure C.** Measured NO_3_^-^ concentration (y-axis) in solutions containing 10, 25, 80 and 200 µM NO_3_^-^ and each containing 0, 1, 5, 10, 50, 100, 400 and 800 µM Fe^2+^. The 1:1 actual:measured NO_3_^-^ concentration is represented by the solid line. Symbols represent all data points (average of 3 experimental replicates) for 200 (black circles), 80 (red squares), 25 (blue triangles) and 10 (pink diamonds) µM NO_3_^-^ concentrations.

**Figure D.** Measured NO_3_^-^ concentration in solutions containing 10, 25, 80 and 200 µM NO_3_^-^ in the presence of 0, 1, 5, 10, 50, 100, 400 and 800 µM Fe^2+^. Dashed horizontal lines represent actual NO_3_^-^ concentrations. Symbols represent measured NO_3_^-^ concentrations (average of three experimental replicates) and error bars their standard deviation (200, circles; 80, squares; 25, triangles; 10, diamonds). (A) shows all of the data; for clarity (B) presents an expanded x-axis with results for 0-10 µM Fe^2+^.

(A)

(B)

**Figure E.** Sequence of reactions involved in the analytical method used for the determination of NO_3_^-^ concentrations.

Note: 1) the reaction occurs under acidic conditions, and 2) in our analytical method, the *reaction vessel* contained 11,930 µM VCl_3_, 4,340 µM sulfanilamide, 144.7 µM *N*-1-napthylethylenediamine, and 50, 20, 6.25 or 2.5 µM NO_3_^-^, with Fe^2+^ concentrations in the range 0.25-200 and 0 µM. The strength of the reagent, as well as the sample:reagent ratio, can be easily modified to accommodate analysis requirements.


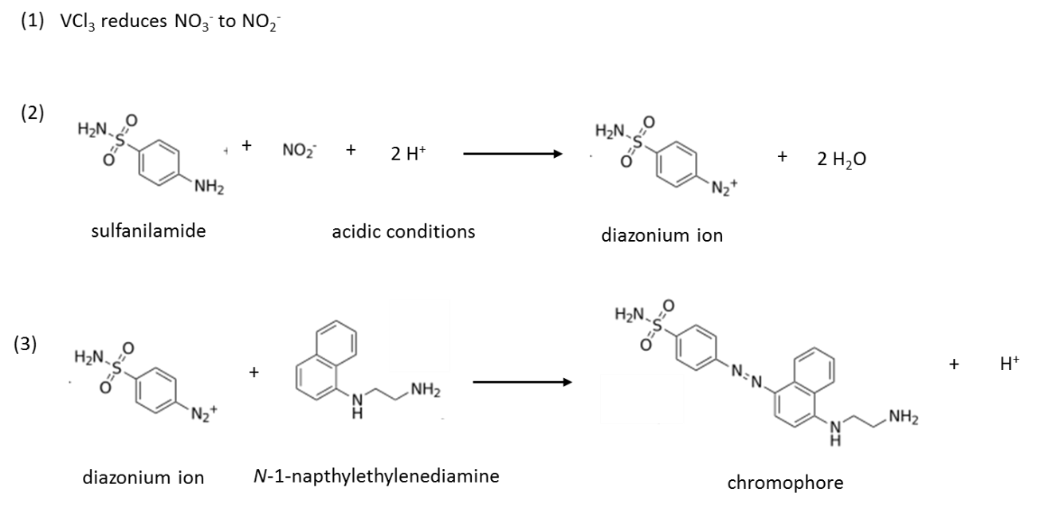


**V. References.**

Berns, A. E.; Philipp, H.; Narres, H. D.; Burauel, P.; Vereecken, H.; Tappe, W., Effect of gamma-sterilization and autoclaving on soil organic matter structure as studied by solid state NMR, UV and fluorescence spectroscopy. European Journal of Soil Science 2008, 59, (3), 540-550.

Colman, B. P.; Fierer, N.; Schimel, J. P., Abiotic nitrate incorporation in soil: is it real? Biogeochemistry 2007, 84, 161-169.

Dail, D. B.; Davidson, E. A.; Chorover, J., Rapid abiotic transformation of nitrate in an acid forest soil. Biogeochemistry 2001, 54, (2), 131-146.

de la Rosa, J. M.; Liebner, F.; Pour, G.; Knicker, H., Partitioning of N in growing plants, microbial biomass and soil organic matter after amendment of N-ammonoxidized lignins. Soil Biology & Biochemistry 2013, 60, 125-133.

Doane, T.A.; Horwáth, W.R. Spectrophotometric determination of nitrate with a single reagent. Analytical Letters, 2003, 36, 2713–2722.

Fry, B., Stable Isotope Ecology. Springer: New York, NY USA, 2006.

Smernik, R. J.; Baldock, J. A., Does solid-state (15)N NMR spectroscopy detect all soil organic nitrogen? Biogeochemistry 2005a, 75, (3), 507-528.

Smernik, R. J.; Baldock, J. A., Solid-state N-15 NMR analysis of highly N-15-enriched plant materials. Plant and Soil 2005b, 275, (1-2), 271-283.

Thorn, K. A.; Cox, L. G., N-15 NMR spectra of naturally abundant nitrogen in soil and aquatic natural organic matter samples of the International Humic Substances Society. Organic Geochemistry 2009, 40, (4), 484-499.

Torres-Cañabate, P.; Davidson, E.A.; Bulygina, E.; García-Ruiz, R.; Carreira, J.A. Abiotic Immobilization of Nitrate in Two Soils of Relic Abies Pinsapo-Fir Forests under Mediterranean Climate, Biogeochemistry, 2008, 91, 1-11.

Yang, W.H.; Herman, D.; Liptzin, D.; Silver, W.L. A new approach for removing iron interference from soil nitrate analysis. Soil Biology and Biochemistry, 2012, 46, 123-128.
